# Health in the Americas 2007. Volume 1 and 2

**Published:** 2008-12

**Authors:** Tracey Perez Koehlmoos

**Affiliations:** Head, Health and Family Planning Systems Programme ICDDR, B

PAHO's new *Health in the Americas 2007* is a tome in two parts. The first part features regional issues whereas the second part gives a detailed description of the state of health and healthcare for each country in the region. Although these oversized texts would not be recommended for light holiday reading, there is plenty to enjoy—particularly for those of us who remember curling up with a good encyclopaedia or are health systems aficionados. Be warned, however, this is far from your grandfather's (or *abuelo's*) dour statistical publication, the new Health in the Americas works hard to stay fresh for the modern reader who is more familiar with accessing numbers and percentages from the Internet.

Volume 1 provides a summary of the regional issues. It contains several excellent chapters that could stand alone as publications and should be made available both in English and Spanish on the Internet as such, especially parts of the chapter entitled *Public Policies and Health Systems and Services*. The chapter on Health in the Context of Development includes well-designed insight into the region's strides towards achieving the Millennium Development Goals as do subsequent chapters. The expected chapter on *Health Conditions and Trends* does more than inform about vaccine uptake and maternal mortality nation by nation; it actually offers something fresh by including new threats to the region by bioterrorism, man-made disasters, the impact of Hurricane Katrina, and the growing regional threat of non-communicable diseases in all the countries facing the ageing populations and the double burden of disease. The *Sustainable Development and Environmental Health* chapter discusses such trendy issues as healthy communities and spaces in addition to the standard yet important features on access to sanitation and clean water. Further, lifestyle-related community risk factors, like alcohol, tobacco use, and traffic accidents, are evaluated. Throughout the text, the plight of the indigenous population in the Americas is brought to the surface and examined. Also, the first volume does an exceptional job of balancing behemoths like the USA, Canada, and Brazil with the smaller and more diverse members of the Latin America and the Caribbean.

Further, inset boxes are used for lists and more importantly to contain definitions. The users of this work would be multidisciplinary and perhaps unfamiliar with the difference between segmentation and fragmentation or indigence and poverty. Perhaps my favourite table describes the comparative advantages and disadvantages of health-sector reform during the last twenty years in Latin America and the Caribbean. The text is also sprinkled with quotations from the likes of Cummings, Horowitz, Alleyne, and Guerra de Macedo, which adds to the sense of historical importance. After all, this is PAHO's flagship publication so a bridge between the past, the present and the future is appropriate to show just how far the region has progressed since PAHO was founded during the last century.

Volume 2, which is the larger of the two books, is a country-by-country compendium of health and health systems information from around the region. It begins with Anguilla, and the regional alphabetical order ends in Venezuela. Each country-section includes a map for those unsure of the exact locations and regional breakdown of Anguilla, Saint Lucia, or any of the 46 countries and territories in the PAHO region. More significantly, each country-study begins with an overview of social, political and economic determinants of health before launching into the country-specific demographics, burden of disease, and health system's response and organization. If looked together, there is a lot of repetition, but from a comparative health systems perspective, there is a lot to glean from this apples-to-apples textual format.

My favourite and perhaps the most interesting chapter in this nation- and territory-based text features the *United States-Mexico Border Area*, which is the concluding chapter in Volume 2. A long way both from Washington, DC and the District Federal, PAHO has long recognized this area as a distinct region in which the populations on two-sides of the border share more in common with one another than with the rest of either of their nations. The impact of diverse policies and regulations across a border of nearly 2000 miles and a common commitment to achieving health goals, make this the chapter that should be read first in this book or second after exploring the chapter on the health system and status of one's own home country.

Overall, there are plenty of charts, maps, and graphs for the more pictorially inclined in both Volume 1 and 2. To that end, Volume 1 does a fine job of showing the information in a varied but subdued colour scheme. However, the use of only black and shades of blue in the second volume creates limitation and uncertainty in displaying complex information. One example of this shortcoming is the chart on *Obesity trends among adults in the United States of America* over a ten-year period.

Far from an atavistic throwback to the days of large, dull publications filled with demographic statistics, PAHO's latest *Health in the Americas* is relevant now and more engaging than ever. Although still overflowing with numbers, figures, and percentages on the expected significant health issues in the region, these volumes have reinvented themselves like the phoenix and, again, it is predicted to change and reappear in 2012 as promised in the Introduction by PAHO Director Mirta Roses Periago. The challenge will be to keep the demographic information up-to-date and available on the Internet and to keep producing excellent volumes that are both relevant and easily accessible in the changing landscape of global health and relevant literature.

